# Diagnosis and management of adrenal incidentaloma: use of clinical judgment and evidence in dialog with the patient

**DOI:** 10.1007/s00595-023-02781-y

**Published:** 2023-12-13

**Authors:** Yusaku Yoshida, Kiyomi Horiuchi, Michio Otsuki, Takahiro Okamoto

**Affiliations:** 1 Departments of Endocrine Surgery, Tokyo Women’s Medical University, 8-1 Kawada-Cho, Shinjuku-Ku, Tokyo, 162-8666 Japan; 2 Departments of Endocrinology, Tokyo Women’s Medical University, 8-1 Kawada-Cho, Shinjuku-Ku, Tokyo, 162-8666 Japan

**Keywords:** Adrenal incidentaloma, Diagnosis, Management, Evidence, Dialog

## Abstract

The prevalence of adrenal incidentaloma (AI) in imaging studies, including those of the adrenal glands, is estimated to be 1–5%. Essential factors for the proper management of AI include a correct diagnosis, adequate surgical skills, appropriate perioperative management, and sound dialogue with the patient. Aside from the possibility of overdiagnosis, patients with apparent signs or symptoms attributable to adrenal hormone excess have reasonable indications for surgery. At the same time, milder patients may be candidates for active surveillance without intervention. Even individuals with nonfunctioning AI may benefit from surgery if imaging studies depict the tumor as suggestive of malignancy. However, a differential diagnosis of AI may not be easy for surgeons with little experience in seeing such patients.

Furthermore, a patient without a correct diagnosis may miss the window of opportunity for a cure or incur a greater risk of developing complications, such as adrenal insufficiency or cardiovascular events during or after surgery, due to inadequate management. The clinical practice guidelines for AI from around the world may be helpful for shared decision-making; however, Japan lacks established guidelines. In this review article, we propose practical guidelines relevant to management by summarizing the evidence for five key questions that are often asked in dialog with patients with AI.

## Introduction

Adrenal incidentaloma (AI) is a tumor of the adrenal gland that is found incidentally during clinical investigations for other purposes. The prevalence of AI is estimated to be 1–5% in scans that include the adrenal glands within the imaging field [1–4]. Detection of an AI can represent an overdiagnosis associated with unnecessary worry, additional costs, and potential harm from subsequent investigations of patients who do not feel *dis-eased* or *dis-ordered* [5]. However, some patients with AI meet definitive indications for surgery. A patient without a correct diagnosis may miss the opportunity to cure or develop complications during or after adrenalectomy owing to improper management. For example, adrenal insufficiency may develop following the resection of a cortisol-producing tumor. In patients with pheochromocytoma, cardiovascular and cerebrovascular complications may become a concern in the intraoperative and postoperative periods [6, 7].

A physician with an opportunity to see a patient with AI should consult with endocrinologists or endocrine surgeons if they are unfamiliar with the condition. However, such help may not be readily available because of limitations on available resources. Several professional and academic societies have developed clinical practice guidelines on the management of AIs, including the National Institutes of Health, the European Society of Endocrinology in collaboration with the European Network for the Study of Adrenal Tumors, the American Association of Clinical Endocrinologists in collaboration with the American Association of Endocrine Surgeons, and the Korean Endocrine Society [3, 8–10]. However, no such guidelines have yet been developed specifically for Japan.

**Fig. 1:**
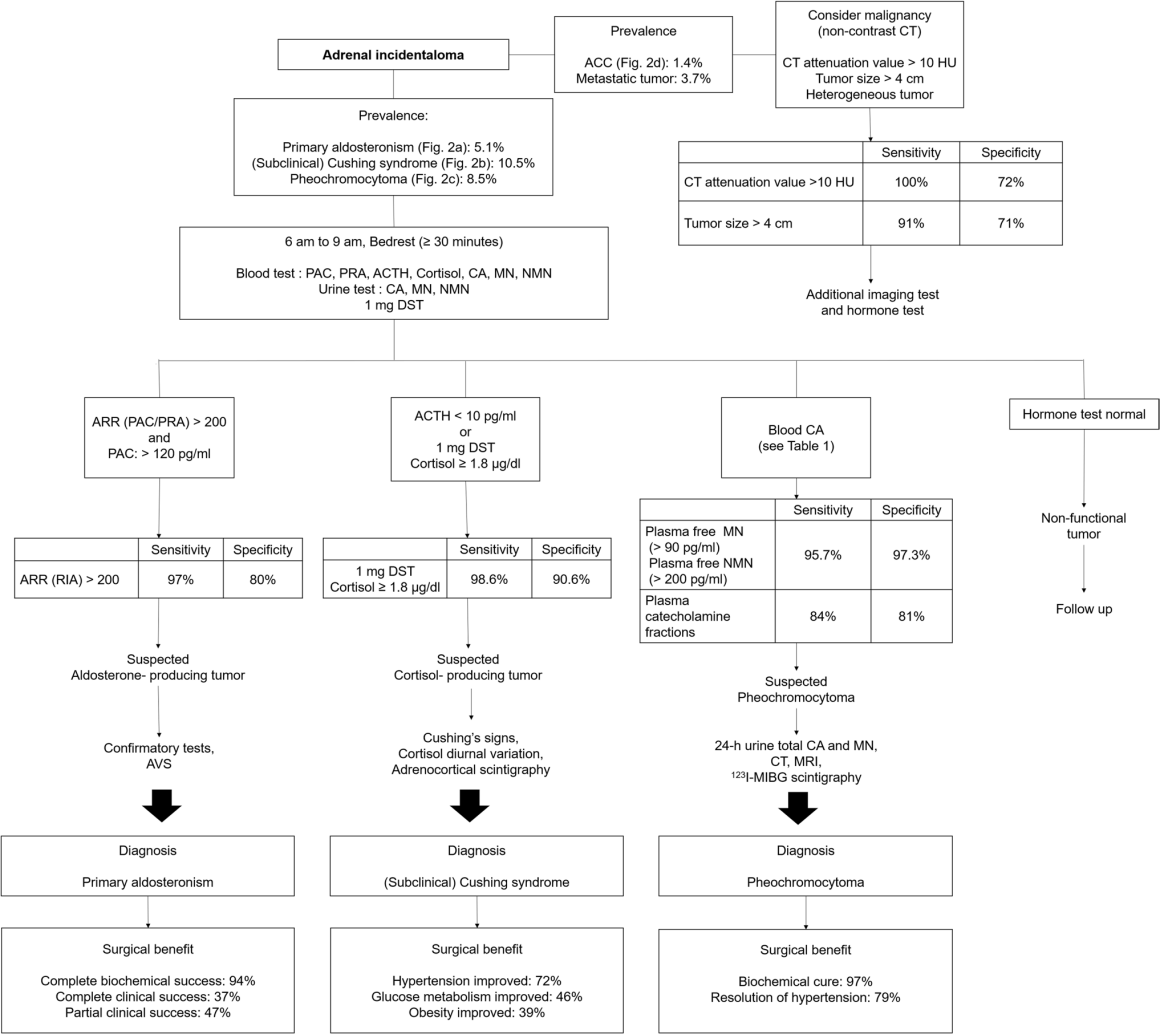
Algorithm of adrenal function screening test

We herein propose an evidence-based approach for patients with AI using five key questions (KQs) relevant to the dialog in clinical practice. First, ‘Do I need further tests?’ Second, "Is this tumor functional?" Third, "Is this tumor benign or malignant?" Fourth, "What are the benefits and disadvantages of adrenalectomy?" Finally, "What will happen if a nonfunctioning tumor or tumor with mild autonomous cortisol excess (MACE) is left untreated?" We adopted a question-and-answer format following previous work by one of the authors [11, 12] while recognizing a recent article on a similar topic by Ceccato et al. [13]. In particular, we provided quantitative summaries (evidence) of the uncertainties inherent to each KQ that is essential for shared decision-making (Fig. 1) [11, 12]. We searched the relevant literature, including the existing guidelines, to identify evidence pertinent to each KQ. We first prioritized systematic reviews, followed by observational studies relevant to KQ, but critically evaluated each piece of evidence in terms of internal and external validity. The probability of a specific diagnosis based on a test was estimated by Bayes' theorem using prevalence data and test performance [14].

### KQ1: Do I need further tests?

#### Evidence for dialog

- Most AIs (75%) are innocent [1].
- Few AIs meet the indications for surgical treatment because of hormone overproduction (25%) or the rare possibility of adrenal cancer (1%) [1].

#### Comments

Physicians must be aware that a detected AI inherently poses a risk of overdiagnosis, leading to unexpected anxiety and cost burdens for the patient [5]. Assuring patients that most AIs are innocent while explaining the importance of additional tests that may clarify the nature of the AI is essential for shared decision-making.

### KQ2: Is this tumor functional?

#### Evidence for dialog

**Fig. 2:**
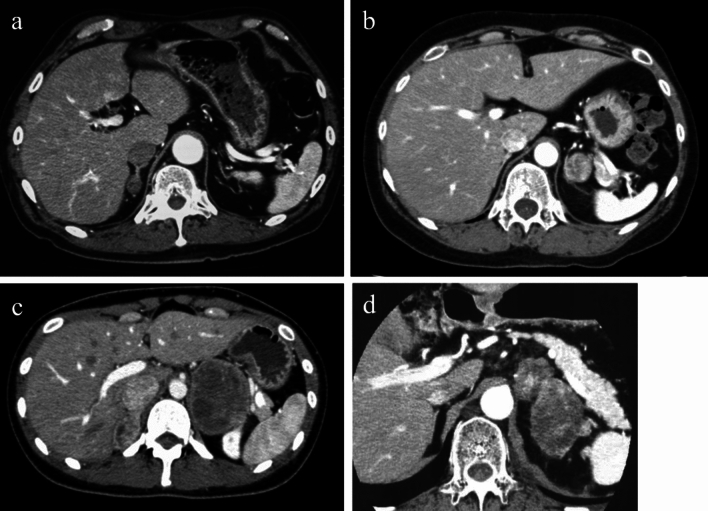
**a** Right adrenal aldosterone-producing tumor. **b** Left adrenal cortisol-producing tumor. **c** Bilateral adrenal pheochromocytoma. **d** Left adrenocortical carcinoma

- The prevalence of functioning AIs in Japan is estimated to be approximately 25%; these consist of cortisol-producing tumors in 10.5% of cases, aldosterone-producing tumors in 5.1%, and catecholamine-producing tumors in 8.5% (Fig. 2a–c) [1].
- The sensitivity and specificity of the plasma aldosterone-to-renin ratio (ARR) with a cutoff value of 200 to screen for primary aldosteronism are estimated to be 97% and 80%, respectively [15].
- The sensitivity and specificity of the overnight 1-mg dexamethasone suppression test (DST) with a cutoff value for plasma cortisol of ≥ 50 nmol/l (1.8 µg/dl) to screen for hypercortisolism were estimated to be 98.6% and 90.6%, respectively [16].
- The sensitivity and specificity of fractionated plasma-free metanephrines for screening pheochromocytoma are estimated to be 95.7% and 97.3%, respectively [17].

#### Summary of the relevant literature

##### Prevalence of hormone-producing tumors among AIs

A nationwide survey in Japan reported that the prevalence of cortisol-producing tumors, aldosterone-producing tumors, and pheochromocytoma among adrenal incidentalomas was 10.5% (95%CI 9.5–11.5%), 5.1% (4.4–5.9%), and 8.5% (7.6–9.4%), respectively [1]. The corresponding figures from another study conducted in the Osaka region of Japan were 11.4% (6.7–18.0%), 9.3% (5.2–15.1%), and 4.7% (1.9–9.4%), respectively [18].

##### Performance of screening tests

Clinical practice guidelines recommend the use of a plasma aldosterone concentration (PAC) to plasma renin activity (PRA) ratio (i.e., ARR) ≥ 200 as a screening test for primary aldosteronism (PA) [3, 10, 19–21]. The sensitivity and specificity of ARR > 200 for the diagnosis of PA are 97% (83–100%) and 80% (75–85%), respectively [15]. The negative predictive value calculated based on the prevalence of PA (5.1%) among AIs in Japan was 99.8%. Since the chemiluminescent enzyme immunoassay (CLEIA) replaced the radioimmunoassay (RIA) for the measurement of PAC in 2021, the cutoff value needs to be reconsidered [21, 22]. The Japan Endocrine Society recommends judging the screening test as positive when the ARR is ≥ 200 with PAC ≥ 60 pg/ml and provisionally positive when the ARR is within the borderline range of 100–200 with PAC ≥ 60 pg/ml until the measurement of PAC by the CLEIA is generalized and its optimal cutoff value is established [21].

A suppressed plasma ACTH level < 10 pg/ml between 08:00 and 09:00 indicated hypercortisolism, even if the cortisol level was within the reference range [23]. Some patients with cortisol-producing tumors may have basal plasma levels of ACTH and cortisol within the reference ranges; therefore, a 1-mg DST is essential for diagnosis. The sensitivity and specificity of 1 mg of DST (cortisol ≥ 1.8 μg/dl) for cortisol overproduction are reported to be 98.6% (96.9–99.4%) and 90.6% (86.4–93.6%), respectively [16]. The negative predictive value based on the Japanese prevalence of Cushing's syndrome among individuals with AI (10.5%) was 99.8%. The 1-mg DST should only be performed after screening for pheochromocytoma because this loading test may cause a hypertensive crisis in patients with catecholamine-secreting tumors [24, 25].

Measuring free metanephrine (MN) and normetanephrine (NMN) levels in plasma is the most accurate screening test for pheochromocytoma. Tanaka et al. set cutoff values as either MN > 90 pg/ml or NMN > 200 pg/ml and reported a sensitivity and specificity of 95.7% (91.4–100%) and 97.3% (93.8–100%), respectively [17]. Determination of plasma catecholamine fractions (adrenaline, noradrenaline, and dopamine) offers an alternative screening test with 84% sensitivity (78–89%) and 81% specificity (78–84%) [26]. Measuring free MN and NMN in a single-voided urine test is another test to screen for catecholamine-producing tumors. Ito et al. estimated the sensitivity and specificity with a positive cutoff value of MN + NMN ≥ 1,000 ng/mgCr as 98% (91.4–99.7%) and 100% (78.2–100%), respectively [27]. Measurement of urine MN is a screening test performed in place of a 24-h urine storage test, and the urinary MN and NMN concentrations were adjusted for creatinine for assessment. The calculation method for the single-voided creatinine-adjusted urine MN and NMN is the single-voided urine MN or NMN (µg/dl) / the single-voided urine creatinine concentration (mg/dl). Such biochemical tests help screen for pheochromocytoma because the negative predictive values exceed 96%, while the associated positive predictive values range from 22 to 100% based on the prevalence in Japan among cases with AI (8.5%) (Table 1).

**Table 1:** Sensitivity and specificity of biochemical tests for diagnosing pheochromocytoma, and positive and negative predictive values calculated from prevalence in Japan [1, 17, 26, 27]

	Sensitivity	Specificity	Positive predictive value	Negative predictive value
Plasma free MN	95.7% (91.4–100%)	97.3% (93.8–100%)	76.7%	99.6%
Urinary fractionated MN	97% (92–99%)	69% (64–72%)	22.5%	99.6%
Plasma CA	84% (78–89%)	81% (78–84%)	29.1%	98.2%
Urinary CA	86% (80–91%)	88% (85–91%)	40.0%	98.5%
Urinary total MN	77% (68–85%)	93% (89–97%)	50.5%	97.8%
Urinary VMA	64% (55–71%)	95% (93–97%)	54.3%	96.6%
Single voided urine MN + NMN	98%	100%	100%	99.8%

*MN* metanephrines; *CA* catecholamines; *VMA* vanillylmandelic acid; *NMN* normetanephrines

(): 95% confidence interval

Functioning adrenal tumors are unlikely when all screening tests yield negative results. However, we must be aware that the negative predictive value decreases as the prior probability increases. Therefore, negative results cannot exclude hormone-producing tumors when a patient shows signs or symptoms relevant to excess adrenal hormone or imaging results that show some characteristics of a functioning tumor.

Further tests are warranted when one or more screening tests return positive results. Patients who are positive on screening tests should undergo at least one of the following tests to diagnose PA: the captopril challenge test, saline infusion test, or the furosemide standing test. For individuals diagnosed with PA, adrenal venous sampling (AVS) is essential to differentiate unilateral PA from bilateral PA when surgery is indicated, for the following reasons. First, > 70% of cases of primary aldosteronism involve both adrenal glands [28, 29]. Second, the tumor demonstrated on imaging studies is not necessarily the one that produces hormones. Agreement on unilaterality between radiological findings and AVS results is 90% (73.5–97.9%) among individuals of < 35 years of age, in comparison to 69% (62.8–73.8%) among those of ≥ 40 years of age [30]. For patients with overt symptoms of Cushing’s syndrome, a positive result from a 1-mg DST is sufficient to confirm the diagnosis. For those lacking typical manifestations, any of the following findings corroborate a diagnosis of subclinical Cushing’s syndrome (SCS) [23], also referred to as MACE: suppressed basal plasma levels of ACTH < 10 pg/ml; loss of diurnal rhythm in serum cortisol levels (≥ 5 μg/dl at 21.00–24.00); unilateral uptake on adrenal scintigraphy; or low serum levels of dehydroepiandrosterone sulfate. Measuring 24-h urine total catecholamines and metanephrines is essential to confirm the diagnosis of pheochromocytoma [31]. In addition to computed tomography (CT) and magnetic resonance imaging, ^123^I-metaiodobenzylguanidine scintigraphy can help detect multiple lesions and distant metastases.

#### Practical points for screening tests

Screening tests should be performed in the early morning, with the patient in a fasted state after having spent at least 30 min lying down at rest to avoid diurnal variations and other factors that may influence hormone secretion. In addition, some medications may influence hormonal evaluations. For example, most antihypertensive drugs (e.g., beta-blockers, mineralocorticoid receptor antagonists, and diuretics) affect PRA and PAC measurements, leading to false-negative or false-positive ARR results. Therefore, advising patients to replace such drugs with calcium channel blockers or alpha-blockers for 3–4 weeks before testing is appropriate. Since glucocorticoid use can affect ACTH and cortisol and make the assessment of the cortical function challenging, consulting with an endocrinologist for testing in such cases is advisable. In addition, patients are advised to refrain from taking acetaminophen, tricyclic antidepressants, levodopa, adrenergic receptor agonists, or antipsychotics, as well as foodstuffs such as caffeine, bananas, and cheese, which may affect the measurement of catecholamines and metanephrines.

### KQ3: Is this tumor benign or malignant?

#### Evidence for dialog

- The prevalence rates of adrenocortical carcinoma and metastatic adrenal tumors among patients with AI are 1.4% and 3.7%, respectively (Fig. 2d) [1].
- The sensitivity and specificity of non-contrast CT attenuation values > 10 Hounsfield units (HU) for differentiating malignant tumors from their benign counterparts are estimated to be 100% and 72%, respectively [32].
- A cutoff tumor diameter of ≥ 4 cm in patients with no prior history of malignancy offers 91% sensitivity and 71% specificity in the differential diagnosis of malignant lesions [33].
- The sensitivity and specificity of *visual analysis* of 18F-fluoro-2-deoxy-D-glucose (^18^F-FDG) accumulation in AI relative to the liver were 91% and 92%, respectively [34].

#### Summary of the relevant literature

The prevalence rates of *clinically diagnosed* adrenocortical carcinoma and metastatic adrenal tumor among AIs are 1.4% (1.0–1.8%) and 3.7% (3.2–4.5%), while those of pathologically proven lesions are 2.9% (2.1–3.9%) and 3.5% (2.6–4.6%), respectively [1]. A clinical diagnosis of malignancy is clear when imaging indicates specific findings such as invasion into neighboring organs or distant metastasis. Other radiological features of malignancy include heterogeneity, irregularity, rough margins, and calcifications. However, evidence of the diagnostic performance of these findings is sparse [33]. Clinical practice guidelines suggest diagnosing AI as benign when the tumor diameter is < 4 cm and the CT attenuation value is ≤ 10 HU, provided that none of the potential features mentioned above are present (Table 2).

**Table 2:** Imaging characteristics suggesting a benign adrenal tumor

NIH conference 2003 [8]	**Non-contrast CT**
	• Tumor size: < 4 cm
	• CT attenuation value: < 10 HU
	**Chemical shift MRI**
	• T2 low intensity, similar to liver
AACE/AAES guideline 2009 [9]	**Non-contrast CT**
	• Tumor size: < 4 cm
	• CT attenuation value: < 10 HU
	**CT with delayed contrast media washout**
	• Contrast media washout ≥ 50%
ESE/ENSAT guideline 2016 [3]	**Non-contrast CT**
	• Tumor size: < 4 cm
	• CT attenuation value: ≤ 10 HU
	**Chemical shift MRI**
	• Loss of signal intensity on out-phase imaging consistent with lipid-rich adenoma
	**CT with delayed contrast media washout**
	• Absolute washout > 60%
	• Relative washout > 40%
	^**18**^**F-FDG-PET**
	• Absence of FDG uptake or uptake less than liver
Korean Endocrine Society guideline 2017 [10]	**Non-contrast CT**
	• Tumor size: < 4 cm
	• CT attenuation value: ≤ 10 HU
	**CT with delayed contrast media washout**
	• Absolute washout > 60%
	• Relative washout > 40%

*NIH* National Institutes of Health; *CT* computed tomography; *MRI* magnetic resonance imaging; *AACE/AAES* American Association of Clinical Endocrinologists/American Association of Endocrine Surgeons; *ESE/ENSAT* European Society of Endocrinology/European Network for the Study of Adrenal Tumors; ^*18*^*F-FDG-PET* fluorodeoxyglucose positron emission tomography

Two systematic reviews on the performance of CT for diagnosing malignancy among AIs have summarized the sensitivity and specificity of non-contrast CT attenuation values with a cutoff value of > 10 HU as 100% (91–100%) and 72% (60–82%) [32], and 100% and 65%, respectively [33]. However, these figures need to be interpreted with caution given the high or unclear risk of bias among the included studies with regard to patient selection, reference standards, flow, and timing [32]. Furthermore, Sabet et al. reported figures based only on a single study without indicating the exact reference [33].

In that systematic review examining the test performance for tumor size in AI patients with no prior history of malignancy, a cutoff value of 4 cm offered 91% sensitivity (82–96%) and 71% specificity (55–83%), with a positive likelihood ratio of 3.1 (2.0–4.9) and a negative likelihood ratio of 0.13 (0.06–0.25) [33].

A meta-analysis of the diagnostic accuracy of ^18^F-FDG positron emission tomography (PET) and PET/CT estimated that the pooled sensitivity and pooled specificity were 91% (88–94%) and 91% (88–94%), respectively [34]. However, these aggregated figures may not be valid because the included studies were heterogeneous in terms of study population, interpretation criteria, cutoff values, and reference standards [34]. Nonetheless, visual analysis of the accumulation of ^18^F-FDG in the AI relative to the liver is the most practical approach for the differential diagnosis. The performance of this measure was estimated to have 91% sensitivity (83–95%) and 92% specificity (86–95%) [34].

Metastatic tumors of the adrenal gland are nonfunctional. On the other hand, more than half of adrenocortical carcinomas produce hormones such as cortisol, aldosterone, and sex hormones [9, 10, 35, 36]. However, hormone measurement other than in screening tests is not recommended unless hormone-producing adrenocortical carcinoma is evident or suspected from clinical findings, as the utility of such measurements in the differential diagnosis has not yet been determined [9, 10, 35, 36].

Recurrence and metastasis are major factors that affect the long-term prognosis of patients who undergo surgery for pheochromocytoma. Several reports have detailed scoring systems to predict the risk of recurrence based on pathological and clinical findings of pheochromocytoma [37–41]. The Japanese Guidelines for the Treatment of Pheochromocytoma and Paraganglioma 2018 recommend evaluation using the Grading System for Adrenal Pheochromocytoma and Paraganglioma (GAPP) as a scoring system [31, 38]. GAPP scores are used to classify low-, intermediate-, and high-grade pheochromocytomas, which are associated with 5-year survival rates of 100%, 66.8%, and 22.4%, respectively.

Although rare, malignant lymphoma may involve both adrenal glands and cause adrenocortical insufficiency. An elevated serum level of soluble interleukin-2 receptor and substantial accumulation of ^18^F-FDG-PET in the AI can provide clues to suspect this diagnosis. However, a tissue biopsy is essential for a definitive diagnosis [42].

#### Practical points

Table 3 shows positive and negative predictive values using prevalence data and estimated diagnostic performance indices as presented in the evidence. The negative predictive value of non-contrast CT attenuation value, tumor diameter, and PET/CT is exceptionally high, while the positive predictive values of the tests range from 4.3% to 13.9%. Therefore, the tumor is likely to be benign when all the test results are negative, whereas it may be malignant when any of the tests are positive, although the possibility would be less than 14%. Nevertheless, it should be noted that the interpretation criteria in a meta-analysis [34] for ^18^F-FDG-PET include a mixture of visual judgment, standardized uptake value (SUV) ratio, and SUVmax, and the cutoff values that have been used are heterogeneous. Further, the Japanese health insurance system does not cover the use of PET/CT for the differential diagnosis of AI. Finally, intravenous contrast agents are contraindicated for CT scans when pheochromocytoma is suggested based on a biochemical assessment, although using low-osmolar agents may be safe [43].

**Table 3:** Positive and negative predictive values based on prevalence of 1.4% as a pre-test probability

	Sensitivity	Specificity	Positive predictive value	Negative predictive value
Non-contrast CT attenuation value > 10 HU	100% (91–100%)	72% (60–82%)	4.8%	100%
Tumor diameter ≥ 4 cm	91% (82–96%)	71% (55–83%)	4.3%	99.8%
Visual analysis of PET/CT	91% (83–95%)	92% (86–95%)	13.9%	99.9%

(): 95% confidence interval

### KQ4: What are the benefits and disadvantages of adrenalectomy?

#### Evidence for dialog

- Surgery for unilateral primary aldosteronism provides complete biochemical success (94%), normal blood pressure (37%), and improved hypertension (47%)[44].
- Surgery for SCS due to adrenal adenoma improved hypertension (72%), glucose metabolism (46%), and obesity (39%) [45].
- Surgery for pheochromocytoma leads to the resolution of hypertension in 79% and a biochemical cure in 97% of cases [46].
- Laparoscopic adrenalectomy is associated with intraoperative and postoperative complications in 2.8% and 1.6% of patients, respectively [47].

#### Summary of the relevant literature

An international cohort study examined the clinical outcomes of adrenalectomy for unilateral PA using explicit definitions of clinical and biochemical success. Complete clinical success (normal blood pressure without antihypertensive medication), partial success (improved hypertension), and a biochemical cure (normalization of ARR) were achieved in 37%, 47%, and 94% of patients, respectively [44]. Benham et al. conducted a meta-analysis on the proportion of patients with resolution of hypertension, defined as normal blood pressure without medication following adrenalectomy in patients with PA. Although the stratified meta-analytic aggregation of PA patients for whom the pathology was limited to unilateral adrenal adenoma estimated the pooled proportion to be 54% (95%CI 41–67%), the studies included were still heterogeneous [48]. Predictors associated with resistant hypertension after adrenalectomy for unilateral PA include male sex, older age, higher levels of preoperative medication, and obesity [49–51]. A systematic review indicated that the effects of mineralocorticoid receptor antagonists (MRA) were comparable to those of adrenalectomy in terms of various outcomes [52]. However, a Japanese study observed that surgery was superior to MRA for improving hypertension and hypokalemia in unilateral PA [53]. In addition, adrenalectomy provided better results than medical treatments for quality of life and psychological symptoms [54–56]. Japanese clinical practice guidelines recommend surgical treatment for unilateral PA to resolve hypertension and achieve positive effects on organ damage [21].

The clinical benefit of surgical treatment for overt Cushing’s syndrome is not controversial but has not been substantiated by outcome data in the relevant literature. A systematic review of observational studies with 10 or more adrenal SCS patients revealed that adrenalectomy improved hypertension in 72% of patients, diabetes mellitus in 46%, and obesity in 39% [45]. Another systematic review of case series studies with five or more adrenal SCS cases reported corresponding proportions of 61%, 52%, and 45% [57]. However, these figures were calculated from simple summations (not meta-analytic aggregations) of numbers from the included studies, which used different SCS criteria.

Once a diagnosis of pheochromocytoma is made, the patient has a definite indication for surgery. In addition to its malignant potential, the disease may result in cardiovascular complications and hypertensive crisis, which are triggered by various physical stressors [58].

A retrospective study found that adrenalectomy achieved resolution of hypertension in 79% and biochemical cure in 97% of 159 patients with pheochromocytoma [46]. An international, multicenter, retrospective study of 1860 patients with pheochromocytoma and paraganglioma treated surgically showed an overall mortality rate of 0.5% and an overall cardiovascular complication rate of 5.0% [59].

Among the 19,534 patients who underwent laparoscopic adrenalectomy in Japan, 549 (2.8%) experienced intraoperative complications and 308 (1.6%) experienced postoperative complications. Furthermore, 300 patients (1.5%) required conversion to open surgery [47]. Two systematic reviews found that the laparoscopic approach was superior to open surgery for pheochromocytoma in terms of stability of intraoperative hemodynamics, blood loss, need for transfusions, postoperative complications, and postoperative hospital stay [60, 61].

#### Practical points

Surgery plays a definitive role in managing patients with functioning adrenal tumors, particularly those with pheochromocytoma and overt Cushing’s syndrome. However, the indications for the surgical treatment of SCS remain controversial. The Japan Endocrine Society suggests surgery for SCS under conditions of either serum cortisol level ≥ 5 mg/dl after a 1-mg DST or tumor diameter ≥ 3 cm [23]. Medical treatment with MRA is an alternative to surgery for managing PA, but its comparative efficacy in terms of long-term outcomes has yet to be clarified.

### KQ5: What will happen if a nonfunctioning tumor or tumor with MACE is left untreated?

#### Evidence for dialog

- The proportions of nonfunctioning tumors and MACE adenomas that increased in size by ≥ 1 cm were 1.2% and 2.4%, respectively, during a mean follow-up period of 50.2 months [62].
- The probability of developing MACE from a nonfunctioning adrenal tumor was estimated to be 4.3%, while that of resolving preexisting MACE was 0% during a mean follow-up period of 50.2 months [62].
- The probability of developing overt Cushing’s syndrome from nonfunctioning adrenal tumors or MACE adenomas was estimated to be 0.7% in one systematic review and 0.2% in another [62, 63].
- Patients with MACE adenomas are more likely to develop or experience an exacerbation of cardiometabolic comorbidities than patients with nonfunctioning adrenal tumors during follow-up [62].
- The probability of malignant transformation was 0% during a mean follow-up period of 49.3 months [62].

#### Summary of the relevant literature

Two systematic reviews of the natural history of AI have been reported [62, 63]. Apart from the years of publication, these two reviews differed in the criteria for the inclusion of individual studies. Of note, Loh et al. limited their study to prospective investigations, while Elhassan et al. allowed both prospective and retrospective studies, yet the designs of the two studies included in the former were judged as retrospective in the latter. On the other hand, the latter analysis did not include two relatively large prospective studies conducted in Sweden [64, 65].

Based on 11 studies with a mean follow-up period of 44.2 months, Loh et al. estimated the pooled incidences of SCS, pheochromocytoma, or overt Cushing’s syndrome as 1.79% (95%CI 0.2–4.5%), 0.41% (95%CI 0.1–0.8%), and 0.7% (95%CI 0.1–1.3%), respectively. Furthermore, the proportion of patients who experienced tumor growth (> 0.5 cm) was 13% (95%CI, 7–21%) [63].

Elhassan et al. included 32 observational studies in which patients with nonfunctioning adrenal tumors (NFATs) or MACE adenomas were followed up without surgery (mean follow-up, 50.2 months). They found that the proportions of NFAT and MACE that increased in size by ≥ 1 cm were 1.2% and 2.4%, respectively. They also estimated that the probability of developing MACE among NFAT patients was 4.3%, while that of resolving preexisting MACE was 0%. Cardiometabolic comorbidities were common among patients with MACE adenomas and NFATs, with estimated prevalence rates of 60%, 42%, 34%, and 18% for hypertension, obesity, dyslipidemia, and type 2 diabetes, respectively. Patients with MACE adenomas were more likely than those with NFAT to show the development or worsening of these conditions during follow-up. None of the 2854 patients with benign NFAT or MACE experienced malignant transformation during a mean follow-up period of 49.3 months. Mortality rates, mainly due to cardiovascular events, were similar between MACE (11.5%) and NFAT (12.0%). The authors pointed out that the main concerns regarding the methodological quality of the included studies were substantial variations in the definitions of MACE and outcomes [62].

#### Practical points

It is imperative to reassure patients with nonfunctioning AI or MACE that the tumor is unlikely to develop clinically significant changes in growth, functional status, or malignant transformation without surgical intervention. According to available observational studies, MACE may be associated with some cardiometabolic conditions, but the causative roles remain unresolved.
